# Supplementary material for: A high-energy sulfur cathode in carbonate electrolyte by eliminating polysulfides via solid-phase lithium-sulfur transformation
Source: Nat Commun. 2018 Oct 30;9:4509. doi: 10.1038/s41467-018-06877-9 (PMC6207722; doi:10.1038/s41467-018-06877-9)
Supplement: Supplementary file 1 — Supplementary Information [file 41467_2018_6877_MOESM1_ESM.pdf]

A high energy sulfur cathode in carbonate electrolyte by eliminating polysulfides via solid-phase lithium-sulfur transformation

Xia Li[1,+], Mohammad Banis[1,2,+], Andrew Lushington[1,+], Xiaofei Yang[1,3], Qian Sun[1], Yang Zhao[1], Changqi Liu[1,3], Qizheng Li[1], Biqiong Wang[1,4], Wei Xiao[1,4], Changhong Wang[1], Minsi Li[1,4], Jianwen Liang[1], Ruying Li[1], Yongfeng Hu[2], Lyudmila Goncharova[5], Huamin Zhang[3], Tsun-Kong Sham[4], and Xueliang Sun[1]*

[1] Department of Mechanical and Materials Engineering, University of Western Ontario, ON, N6A 5B9, Canada E-mail: xsun9@uwo.ca.

[2] Canadian Light Source, 44 Innovation Boulevard, Saskatoon, SK, S7N 2V3, Canada.

[3] Division of Energy Storage, Dalian Institute of Chemical Physics, Chinese Academy of Sciences, Dalian 116023, China

[4] Department of Chemistry, University of Western Ontario, ON, N6A 5B9, Canada.

[5] Department of Physics and Astronomy, University of Western Ontario, London, Ontario N6A 3K7, Canada

+These authors contributed equally to this work.

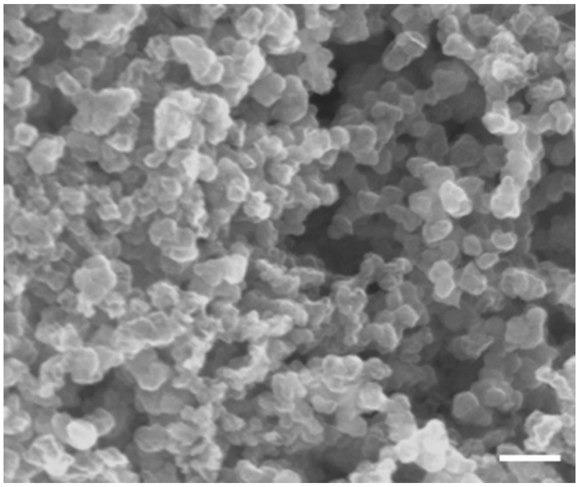

Supplementary Figure 1. FE-SEM image of commercial carbon-sulfur electrode with scale bar 100 nm.

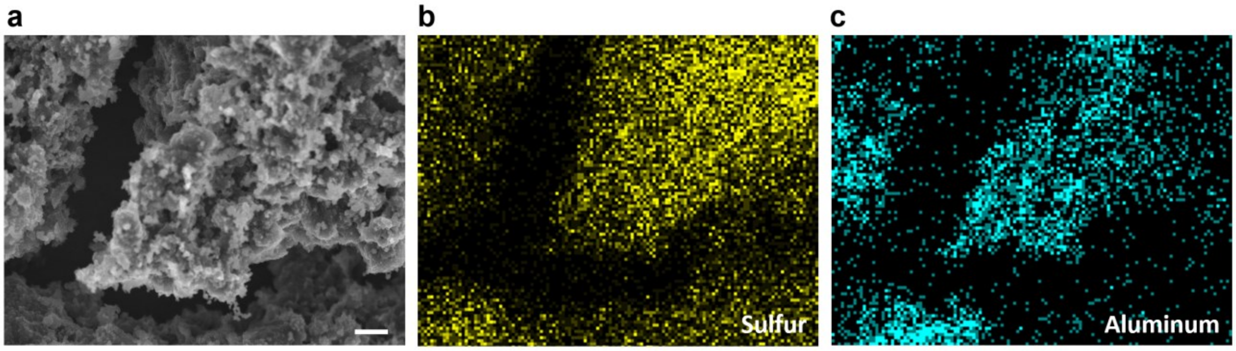

Supplementary Figure 2. FE-SEM image and corresponding element mapping of alucone coated C-S electrodes in a scale bar of 1 μm.

Supplementary Table 1. Summary of reported Li-S batteries in carbonate electrolyte

Name/References	Sulfur content (wt%) Composite/Electrode	Sulfur loading (mg cm^{-2})	Electrolyte	Performance (1st/cycled, mAh g^{-1})	Estimated energy density (Wh kg^{-1}, based on 1st capacity)
Microporous carbon-sulfur composites[1]	42/29.4	1.47 (Sulfur)	$LiPF_6$ (PC:EC:DEC)	1150/745 over 100 cycles	225.66
S_2-S_4 molecules[2]	40/32	1 (active material) /0.40(sulfur)	$LiPF_6$ (EC:DEC)	1670/1190 over 200 cycles	168
Microporous-mesoporous carbon-sulfur[3]	43/34.4	Not mentioned	$LiPF_6$ (EC:DMC:EMC)	1000/900 over 50 cycles	No value
Sulfurized polyacrylonitrile composite[4]	42/35.7	5(active material) /2.1 (Sulfur)	$LiPF_6$ (EC:DEC)	900/680 over 80 cycles	199.29
MOF-derived microporous carbon-sulfur composites[5]	43/34.4	Not mentioned	$LiPF_6$ (EC:DEC)	1400/500 over 100 cycles	No value
Microporous carbon–sulfur composite[6]	40/32	Not mentioned	$LiPF_6$ (PC:EC:DEC)	2000/720 over 100 cycles	No value
Sulfurized polyacrylonitrile composite[7]	50/35	1.4 (SPAN)/0.7 (Sulfur)	$LiPF_6$ (EC:EMC:DMMP)	900/700 over 50 cycles	121.5
Ordered Microporous Carbon Confined sulfur[8]	40/32	1 (Active material) /0.4 (Sulfur)	$LiPF_6$ (EC:DMC)	1600/600 over 500 cycles	160.95
Microporous carbon-sulfur composites[9]	25/20	1 (Active material) /0.25 (Sulfur)	$LiPF_6$ (EC:DEC)	1200/850 over 500 cycles	81.05
Copper-Stabilized Sulfur-Microporous Carbon[10]	50/40	1 (Active material) /0.5 (Sulfur)	$LiPF_6$ (EC:DEC)	1050/630 over 500 cycles	126.21
Sulfur/microporous carbon composites[11]	50/35	Not mentioned	$LiPF_6$ (PC:EC:DEC)	900/520 over 180 cycles	No value
Carbonized Polyacrylonitrile-SeS_x Cathodes[12]	23.1/18.4	1.2(Electrode) /0.22 (Sulfur)	$LiPF_6$ (EC:DEC)	1200/780 over 1200 cycles	66.85
PAN-sulfur composites[13]	45.6/31.9	0.85 (SPAN) /0.38 (sulfur)	$LiPF_6$ (EC:DEC)	1843/1000 over 1000 cycles	151.98
Amorphous S-rich $S_{1-x}Se_x$/C (x< 0.1) composites[14]	47.5/33.25	0.8-1.5 (Active material) /0.38-0.71 (sulfur)	$LiPF_6$ (EC:DMC)	1600/1090 over 200 cycles	196.79
Confined sulfur in microporous carbon[15]	31/21.7	0.17 (Sulfur)	$LiPF_6$ (EC:DEC)	650/500 over 4000 cycles	33.27
Sulfur Confined in Sub-Nanometer-Sized 2D Graphene Interlayers[16]	33.9/27.12	1 (Sulfur)	$LiPF_6$ (EC:DMC:DEC)	1600/600 over 120 cycles	254.06

Sulfur Confined in Sub-Nano porous carbon[17]	30/21	Not mentioned	$LiPF_6$ (EC:DEC)	1600/800 over 100 cycles	No value
Sulfurized polyacrylonitrile[18]	41.8/25	1.5 (Sulfur)	$LiPF_6$ (PC:EC:DEC)+$LiSiO_3$	650/450 over 100 cycles	94.34
S@pPAN composites[19]	44.1/35.28	1.5-2 (Electrode) /0.52-0.72(Sulfur)	LiODFB (EC:DMC:FEC)	1600/1410 over 600 cycles	200.74
Sulfur confined in nitrogen-doped microporous carbon[20]	50/40	1.2-1.4 (Sulfur)	$LiPF_6$ (EC:DMC)	1380/1002 over 200 cycles	270.66
Microporous carbon-sulfur composites[21]	42.5/34	1 (Sulfur)	$LiPF_6$ (EC:DMC)	1200/968.4 over 50 cycles	202.58
Ultramicroporous Carbon-sulfur composites[22]	39.72/28	0.8 (Sulfur)	$LiPF_6$ (EC:DMC:DEC)	800/776 over 200 cycles	115.83
Ultra-microporous carbons-small sulfur composites[23]	37.7/30.1	1 (Sulfur)	$LiPF_6$ (EC:DEC)	1500/852 over 150 cycles	245.44
Fiber-Based Sulfurized Poly(acrylonitrile)[24]	46/32.2	0.672 (Sulfur)	LiTFSI(FEC:DOL)	1100/850 over 1000 cycles	151.03
Alucone coated C-S electrodes[25]	65/45.5	0.9 (sulfur)	$LiPF_6$ (EC:DEC:EMC)	1000/661 over 50 cycles	180.15
Alucone coated C-S electrode	**67/53.6**	**4.0 (Sulfur)**	$LiPF_6$ (EC:DEC:FEC)	1187/705 over 300 cycles	348.27 Estimation of coin cell result
	**65/58.5**	**2.8-3.0 (sulfur)**	$LiPF_6$ (EC:DEC) (E:S= 2.7:1)	1100/480 over 5 cycles	206.30 Actual Pouch cell measurement
					275.23 *Li= 150% of sulfur (Estimated value)
					283.88 *Li=100% of sulfur (Estimated value)

* In the actual pouch cell, the Li foil is thick and the amount of Li is over 800% of sulfur. If using thinner Li foil that 100%-150% of sulfur (the same condition in estimation), the energy density will increase a lot.

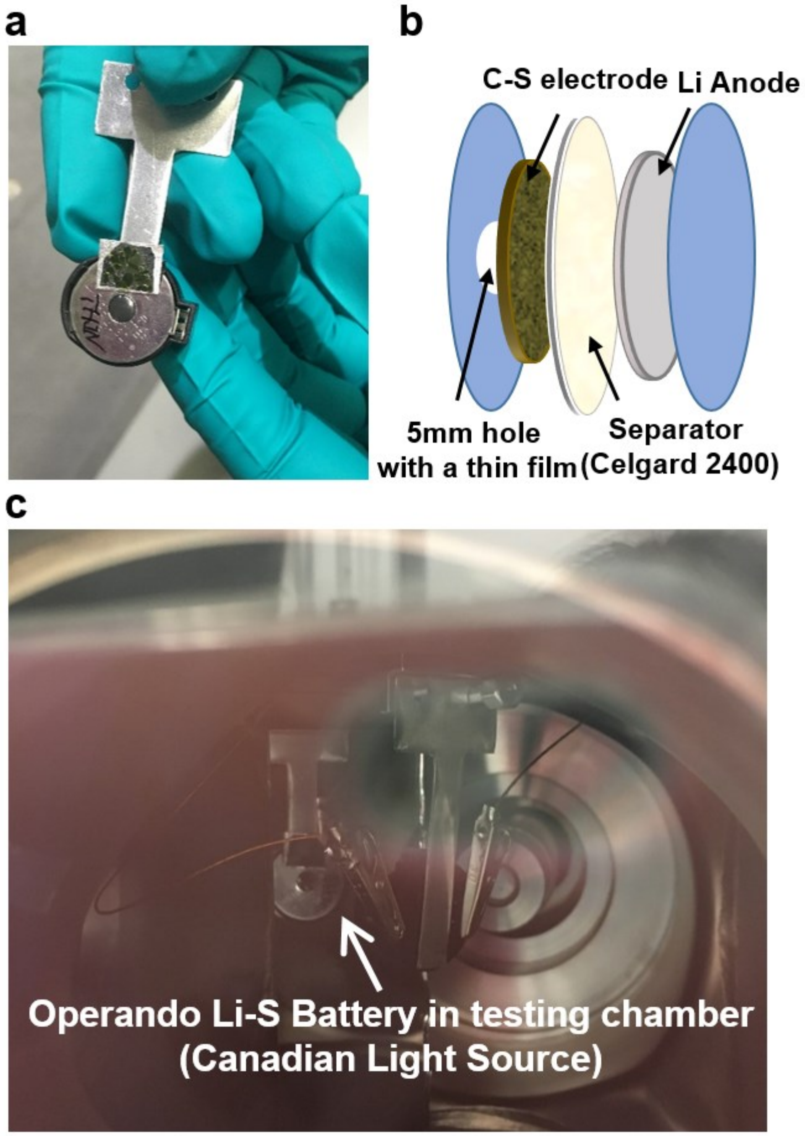

Supplementary Figure 3. (a) Images of an operando Li-S battery with (b) its schematic diagram; and (c) image of the as-prepared Li-S batteries in the testing chamber.

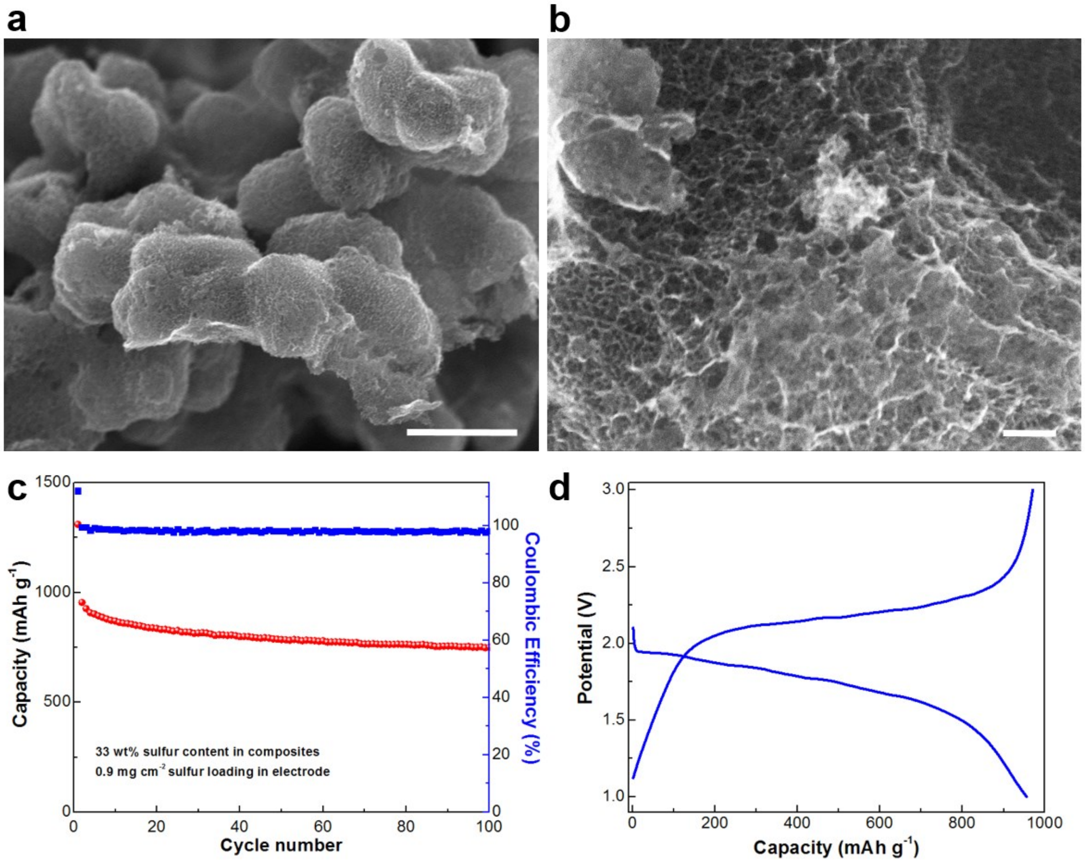

Supplementary Figure 4. Physical and electrochemical characterizations of as-prepared short-chain sulfur cathode materials: (a, b) SEM images of as-prepared microporous carbon hosts. The scale bar are 1μm and 100 nm, respectively; (c) cycle performance of short-chain sulfur cathodes tested at a current density of 160 mA g^{-1}; (d) typical discharge-charge profiles.

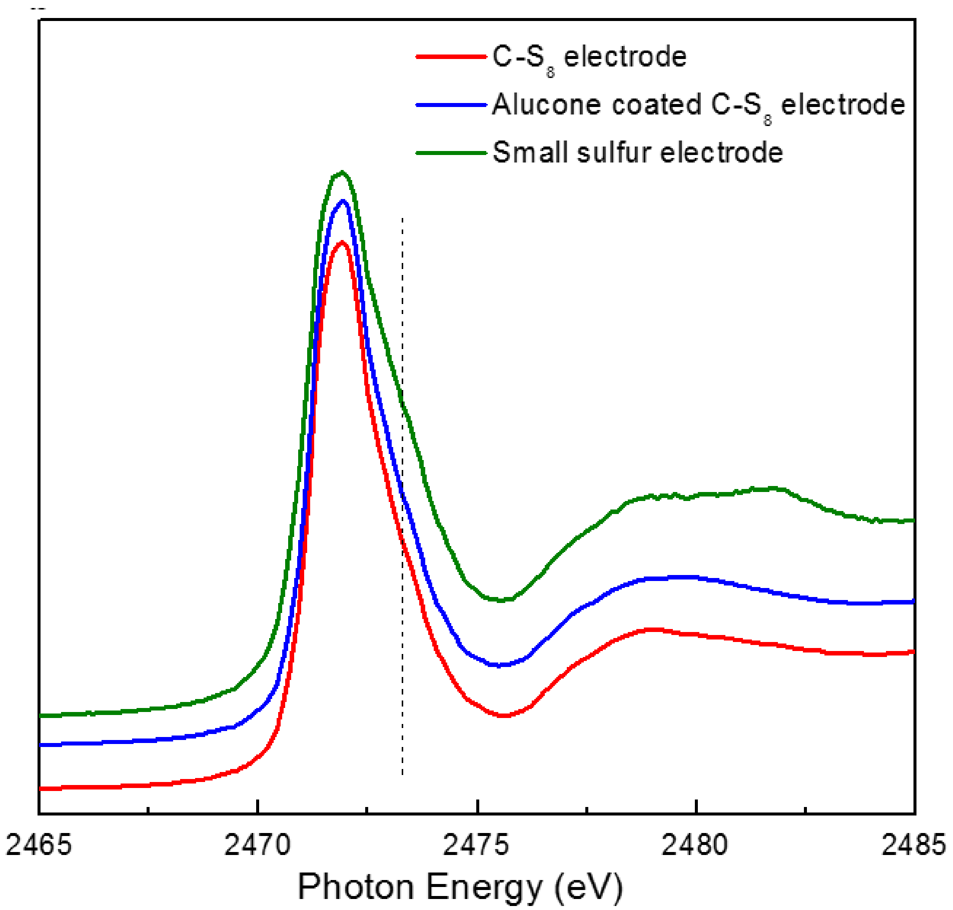

Supplementary Figure 5. X-ray absorption near-edge spectra of the as-prepared sulfur electrodes before electrochemical test.

Compared with the cyclo-S_8 electrode, the small sulfur electrode presents a higher shoulder at 2473.5 eV, which can be assigned to the transition from S 1s to the C-S σ* state. The appearance of this shoulder peak indicates a strong interaction between short-chain sulfur and carbon hosts.

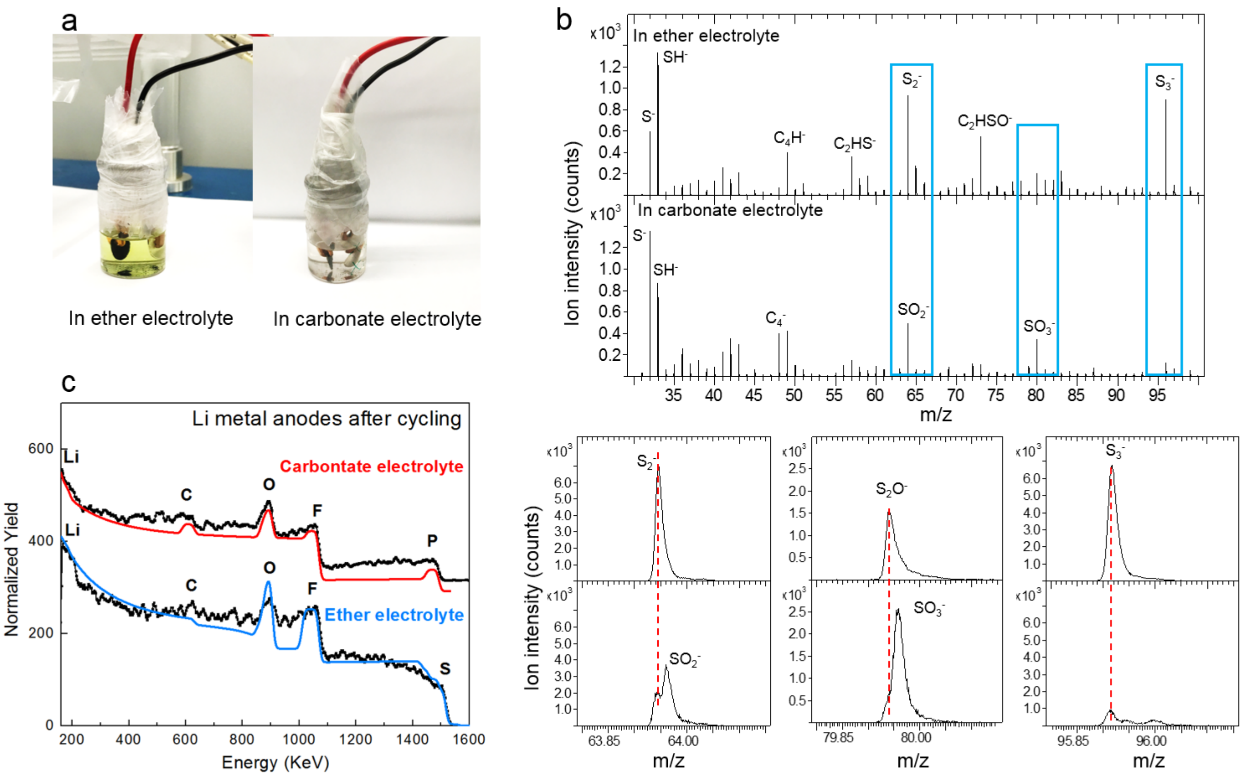

Supplementary Figure 6. Physicochemical characterizations to demonstrate the elimination of polysulfides. (a) Electrolyte color test of the Li-S cell with alucone coated C-S electrodes in ether and carbonate electrolytes. (b) TOF-SIMS spectra of cycled alucone coated C-S electrodes in ether and carbonate electrolytes. (c) RBS spectra of the Li metal anodes in different electrolytes after cycling.

Electrolyte color evaluation of Li-S batteries with alucone coated C-S electrodes is performed in Supplementary Figure 6a. During the electrochemical reaction, the ether-based electrolyte with alucone C-S has changed to light yellow while the carbonate based electrolyte remains constant as colorless liquid, illustrating the elimination of dissolved polysulfides in carbonate based electrolyte. Supplementary Figure 6b presents time of flight secondary ion mass spectra (TOF-SIMS) of the alucone coated C-S electrodes cycled in ether-based and carbonate-based electrolytes. The unique mass fragments are highlighted and shown in the magnified diagrams. From the negative TOF-SIMS spectra, the alucone coated electrode cycled in ether-based electrolyte presents strong peaks of S^-, S^{2-}, S^{3-} species, indicating the formation of linear polysulfides in the electrochemical process. On the other hand, the electrode cycled in carbonate electrolyte only presents the peak of S^- but the peaks of S^{2-} and S^{3-} are not obvious, further demonstrating the absence of polysulfides during the electrochemical process in carbonate electrolyte. To further confirm that no polysulfides diffused and migrated in the battery, Rutherford backscattering spectrometry (RBS) was

performed of the Li metal anodes, as shown in Supplementary Figure 6c. Obviously, the presences of S peak (blue line) confirms the deposition of polysulfide species on Li anode cycled in ether-based electrolyte. However, the spectrum of Li anode cycled in carbonate-based electrolyte (red line) does not show S peak, indicating no dissolved polysulfide deposited on Li metal anode. All of these supporting characterizations further confirm the elimination of polysulfides in the discussed Li-S batteries with carbonate-based electrolyte.

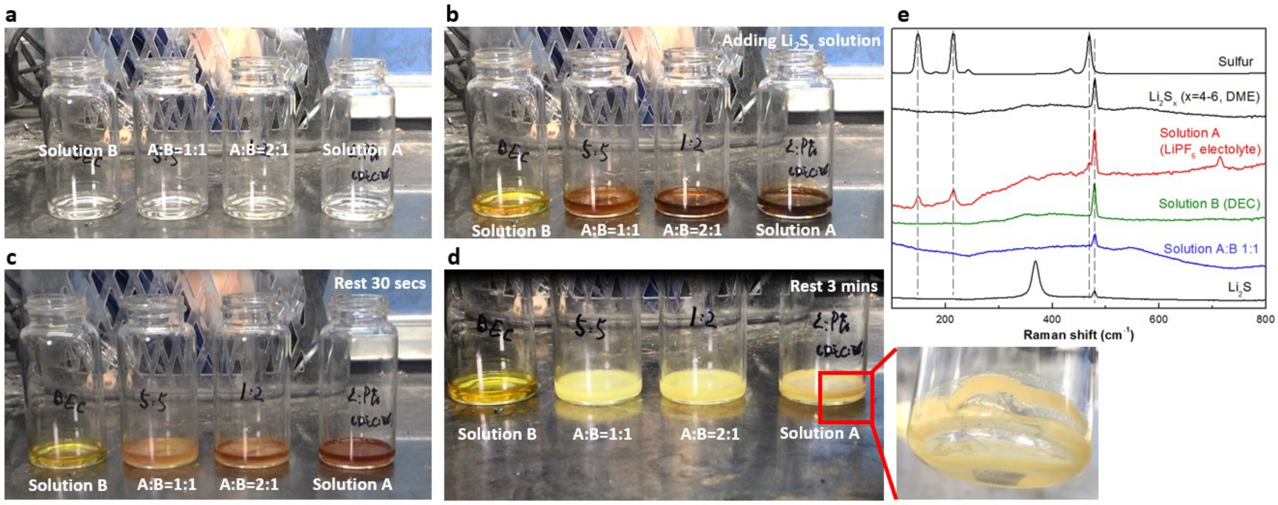

Supplementary Figure 7. Solubility evolution of polysulfides in carbonate based solutions. (a-d) Evolution of different carbonate based liquid with the added drops of polysulfides solution. (e) Raman spectra of the carbonate solutions with polysulfides.

To illustrate the solubility of polysulfides in carbonate electrolytes, we prepared the solutions with 1M $LiPF_6$ in DEC and EC solvent (Solution A); pure DEC solvent (Solution B); and two different mixing ratios with solution A and B (A: B= 2:1 and 1:1) to obtain the different concentration of $LiPF_6$ salt in the carbonate solvents, as shown in Supplementary Figure 7a. With 5-7 drops of polysulfide solution (Li_2S_x, $x \leq 6$, DME solvent), the four solutions experienced different changes, as shown in Supplementary Figure 7b-d. For pure DEC solvents (solution B), the polysulfide solution easily mixed in it and no precipitate formed. For the carbonate solutions mixed with $LiPF_6$ salt, the polysulfide solution is hard to mix with it. In the carbonate solution with a high concentration of $LiPF_6$ (1M, solution A), a large amount of yellow precipitate was formed in a short time (Supplementary Figure 7b-d). Raman characterization was carried out to probe the precipitate materials, as shown in Supplementary Figure 7e, which indicates that the yellow material formed in the solutions is elemental sulfur. These experiments led us to the following conclusion: lithium polysulfides are highly instable when co-existed with $LiPF_6$ based carbonate electrolyte in sharp contrast to merely DEC carbonate solvent, where side reaction occurs with the formation of elemental sulfur as a product.

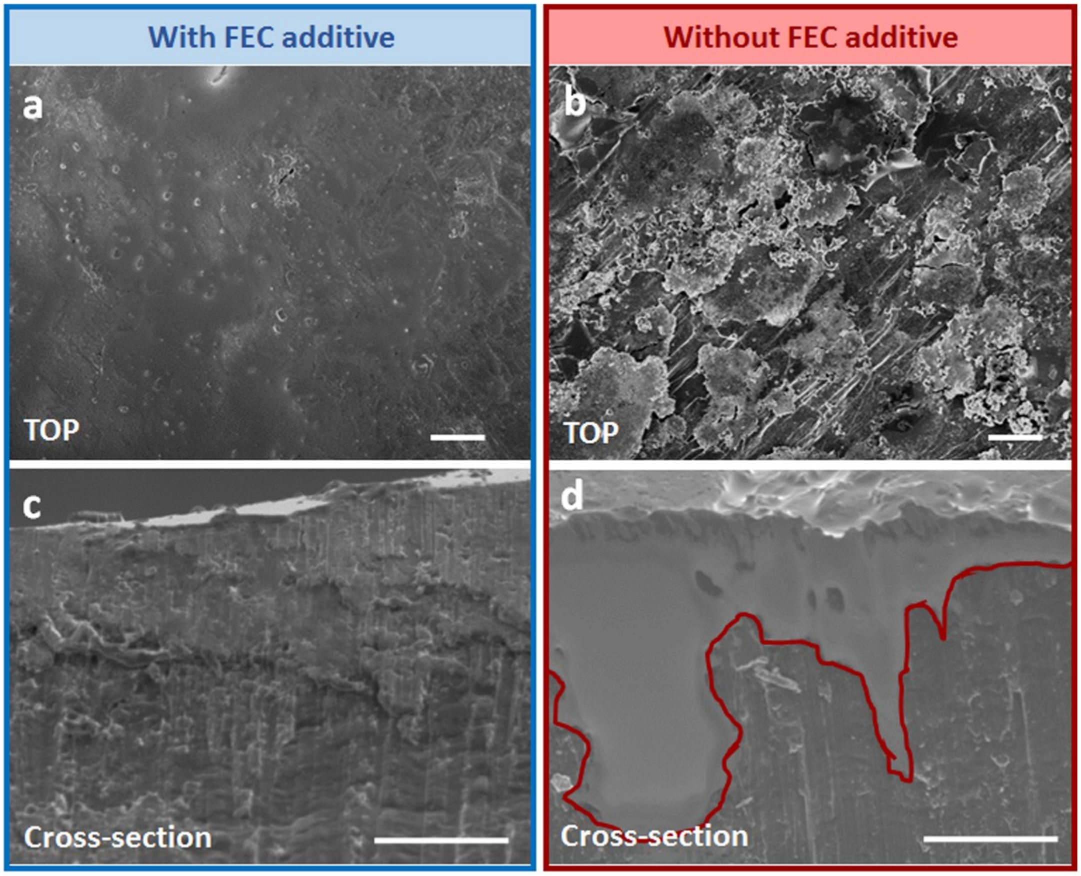

Supplementary Figure 8. Top and vertical view of Li foils operated in carbonate based Li-S cells after 100 discharge-charge cycles (a, c) with FEC additives and (b, d) without FEC additives. The four scale bars in images are 100 μm.

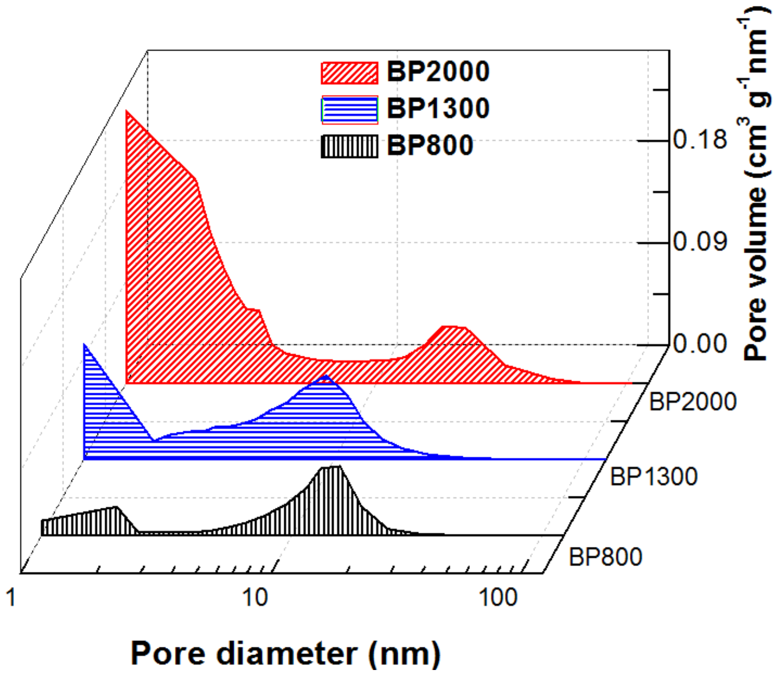

Supplementary Figure 9. Pore size distribution of different carbon hosts.

Supplementary Table 2. Surface properties of employed commercial carbon materials

Carbon	Surface area (m^2 g^{-1})	Micropore volume (cm^3 g^{-1})	Mesopore volume (cm^3 g^{-1})
BP-800	240 m^2/g	0.02	0.65
BP-1300	620 m^2/g	0.10	0.74
KJ EC-600	1020 m^2/g	0.06	0.97
BP-2000	1360 m^2/g	0.24	1.09

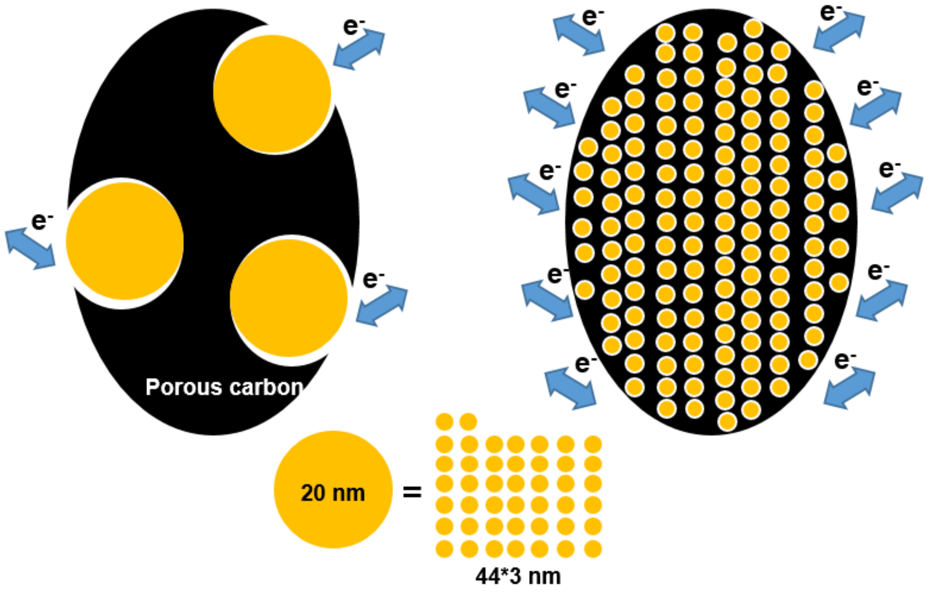

Supplementary Figure 10. Schematic figure of sulfur distribution in different porous carbon hosts.

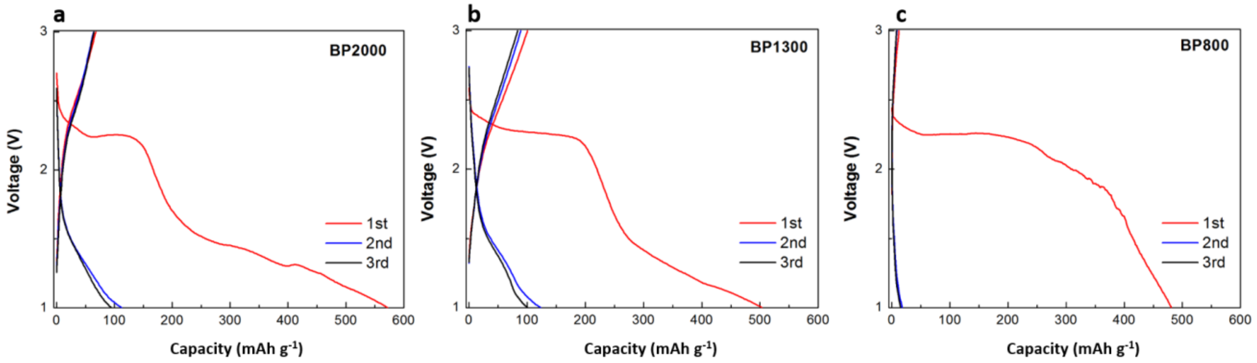

Supplementary Figure 11. Discharge-charge curves of sulfur cathodes without alucone coating in carbonate electrolytes.

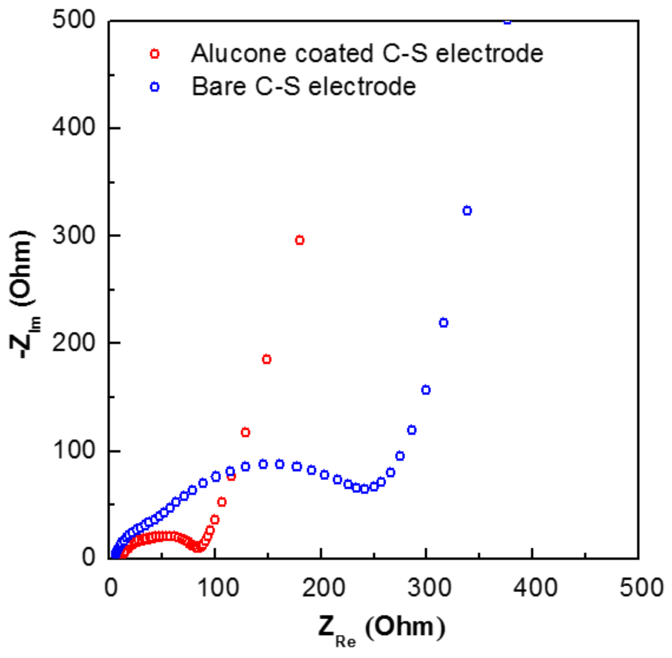

Supplementary Figure 12. EIS plots of the Li-S batteries with different sulfur cathodes after cycling.

Supplementary Figure 12 demonstrates the EIS spectra of the Li-S batteries with different sulfur cathodes cycled in carbonate electrolyte. After twenty discharge-charge cycles in carbonate electrolyte, the battery using alucone coated C-S electrode still maintained very small surface charge transfer resistance, which indicates good conductivity of as-prepared sulfur cathode. However, the battery with bare C-S electrode experienced very large resistance after one discharge-charge cycle.

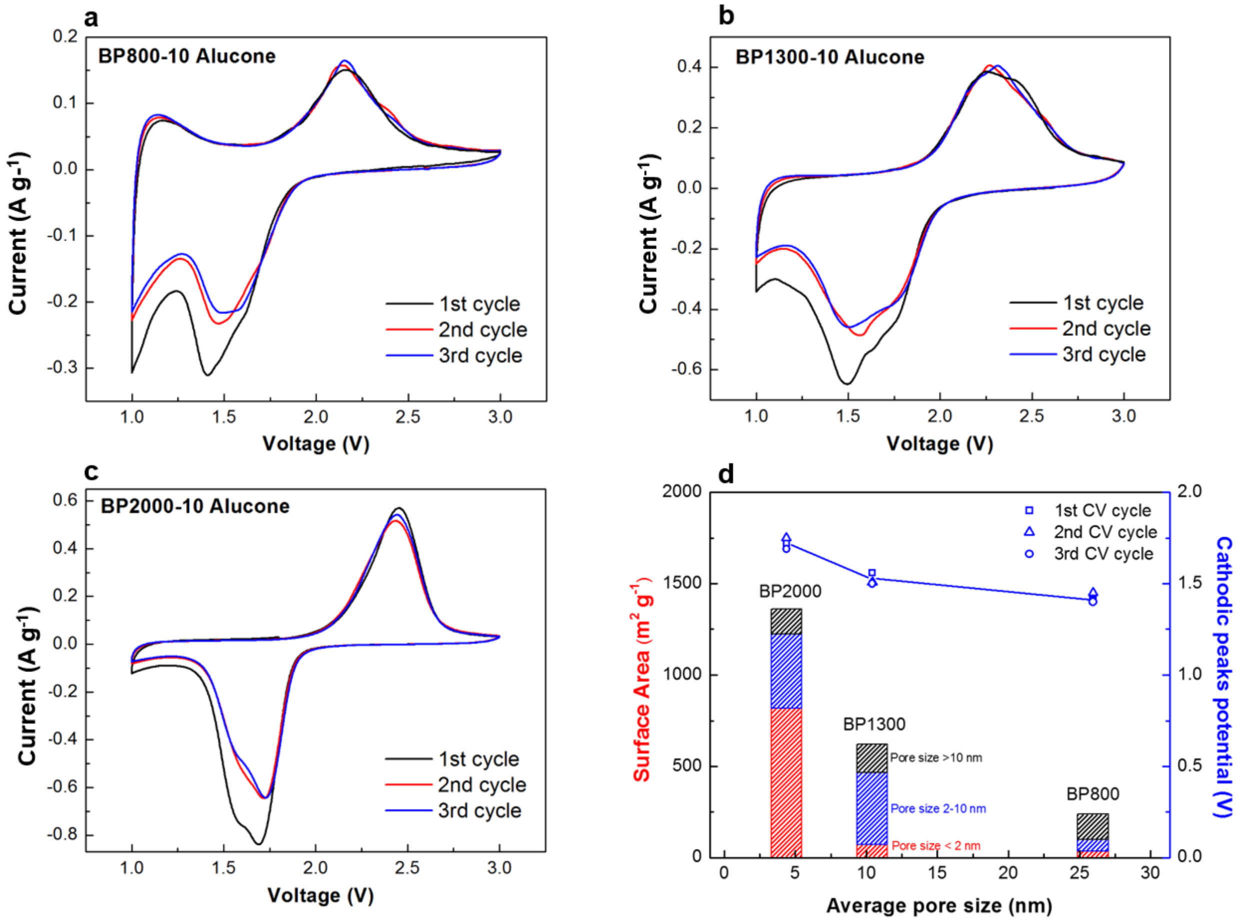

Supplementary Figure 13. Effect of porous structure on the performance of Li-S batteries in carbonate electrolyte. (a-c) CV profiles of the Li-S batteries with different carbon hosts. (d) Summary of the three carbon hosts in terms of surface area, average pore size, and corresponding cathodic peaks of sulfur cathodes in CV profiles.

Supplementary Table 3. Summary of reported Li-S batteries at high and low temperature

	**Cycle performance (Discharge capacity)**	**Temperature**	**Electrolyte**	**Sulfur load**	**Refs**
1.	650 mAh g^{-1}/10th cycle at 0.1 C (only 10 cycle)	45 ^{o}C	LiTFSI (DOL:DME:BTFE) Additive: $LiNO_3$	70 wt%	26
2.	250 mAh g^{-1}/50th cycles at 0.1 C	45 ^{o}C	LiTFSI (DOL:DME) Additive: $LiNO_3$	68 -75 wt%	27
3.	250 mAh g^{-1}/150th cycles at 2 C	70 ^{o}C	LiTFSI (DOL:DME) Additive: $LiNO_3$	1.75 mg/cm^2	28
4.	450 mAh g^{-1}/80th cycles at 1 C	60 ^{o}C	LiTFSI (DOL:DME)	66 wt%	29
5.	400 mAh g^{-1}/100 cycles at 0.2 C	60 ^{o}C	LiTFSI (DOL:DME)	60 wt%	29
6	650-700 mAh g^{-1}/1000 cycles at 850 mA g^{-1}	45 ^{o}C	$LiPF_6$ (FEC:DMC)	<40 wt%	30
7	570 mAh g^{-1}/300 cycle at 0.1 C	55 ^{o}C	$LiPF_6$ (EC:DEC:EMC)	65 wt%	25
8	1010 mAh g^{-1}/200 cycles at 0.1 C	55 ^{o}C	$LiPF_6$ (EC:DEC:FEC)	65 wt%	**Our work**
9	164mAh g^{-1}/ 80 cycles at 1 C	-40 ^{o}C	LiTFSI (DOL:DME)	66 wt%	31
10	800 mAh g^{-1}/1st cycle at 10 mA g^{-1} (Only one cycle)	-10 ^{o}C	$LiN(CF3SO_2)_2$ (TEGDME:DOL:MA)	60 wt%	32
11	380 mAh g^{-1}/200 cycles at 0.1 C	-20 ^{o}C	$LiPF_6$ (EC:DEC:FEC)	65 wt%	**Our work**

Supplementary Table 4. Pouch cell assembly parameters

**Electrode size**	77 mm × 50 mm
**Sulfur host**	BP2000
**Sulfur content**	> 65 wt%
**Sulfur loading (After MLD)**	1.35-1.45 mg cm^{-2} (Single-sided)<br>2.65-2.95 mg cm^{-2} (Double-sided)
**C-S composite: Binder**	9: 1
**Current collector**	Al foil
**Electrolyte**	$LiPF_6$ (EC: DEC, v: v=1: 1)
**Electrolyte: Sulfur**	< 3:1

Estimated practical energy density calculation formula based on a Li-S battery cell without any package:

$$M_{\text{Energy density}} = \frac{E * Q * m_S}{m_{\text{Total}}}$$

Where E is the average discharge voltage, Q is specific discharge capacity based on sulfur, m_S is the sulfur loading, m_{Total} is the total weight of Li-S cell listed in Supplementary Table 5.

Supplementary Table 5. Simulated components of Li-S soft package.

**Components**	**Mass (mg cm^{-2})**
Cathode current collector (Aluminum foil,16 μm)	4.32
Separator (Celgard 2400) [a]	0.9
Sulfur [b]	X
Whole cathode electrode [c]	X/Y
Anode (Lithium metal) [d]	0.65X
Electrolyte [e]	3X
**M_{Total}**	5.22+X/Y+3.65X

[a] The areal density of each layer of Clegard 2400 is 0.9 mg cm^{-2}.

[b] Slurry coated on one sides of cathode current collector with a sulfur loading of X mg cm^{-2}.

[c] Mass ratio of carbon to sulfur in the S/C composite is Y.

[d] 50 wt.% lithium excess accords to the stoichiometric ratio of sulfur.

[e] Mass ratio of electrolyte to sulfur is 3.

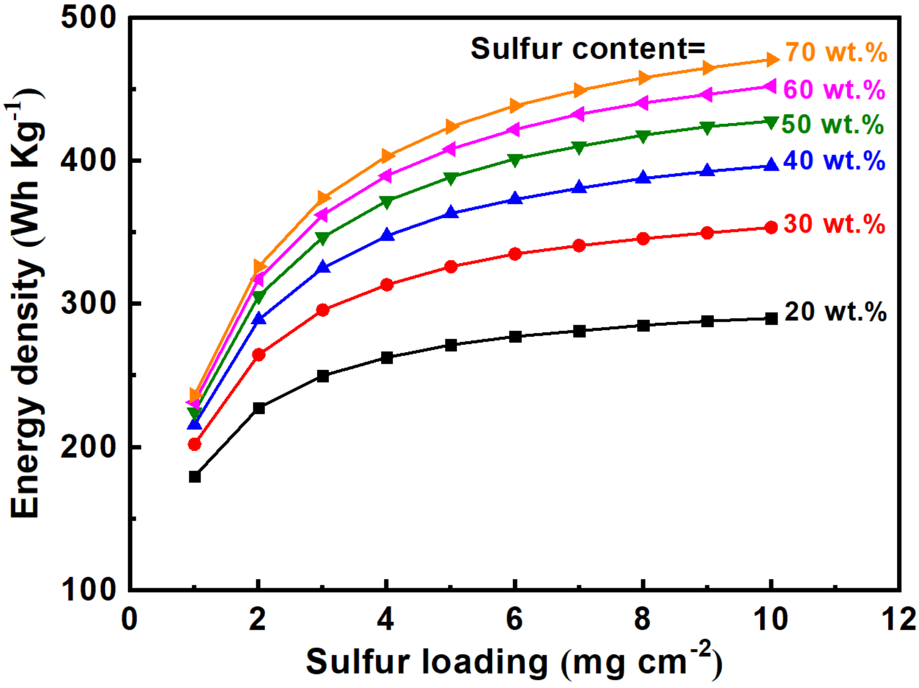

Supplementary Figure 14. Estimation of practical energy density of Li-S batteries in soft package. Energy density calculated based on discharge capacity of 1300 mAh g^{-1} and average discharge voltage of 2.1 V as a function of sulfur content for sulfur loading.

The energy density of Li-S batteries is calculated based on aforementioned formula in which E is 1300 mAh g^{-1} and Q is 2.1 V. This figure demonstrates that the effect of sulfur loading on the battery practical energy density in terms of sulfur content. The result indicates that the high energy Li-S batteries needs both high sulfur content and sulfur loading. For example, if with a low sulfur content (20 wt%), even the sulfur cathode has a high sulfur loading (10 mg cm^{-2}) and excellent performance (1300 mAh g^{-1}), the energy density of battery still cannot meet 300 Wh kg^{-1}. Therefore, for most of reported carbonate based Li-S batteries listed in Table 1, the batteries will have very low practical energy density if applied in soft package with low sulfur content (< 40 wt%) and limited sulfur loading (< 2 mg cm^{-2}). Thereby, the alucone coated C-S electrode with both high sulfur content and high loading applied in carbonate electrolyte has the practical value of Li-S batteries.

Supplementary References

1. Zhang, B., Qin, X., Li, G. R., Gao, X. P. Enhancement of long stability of sulfur cathode by encapsulating sulfur into micropores of carbon spheres. *Energy Environ. Sci.* **3**, 1531 (2010).

2. Xin, S. *et al.* Smaller sulfur molecules promise better lithium-sulfur batteries. *J. Am. Chem. Soc.* **134**, 18510-18513 (2012).

3. Wang, D. W.*, et al.* A microporous-mesoporous carbon with graphitic structure for a high-rate stable sulfur cathode in carbonate solvent-based Li-S batteries. *Phys. Chem. Chem. Phys.* **14**, 8703-8710 (2012).

4. Wang, L., He, X., Li, J., Chen, M., Gao, J., Jiang, C. Charge/discharge characteristics of sulfurized polyacrylonitrile composite with different sulfur content in carbonate based electrolyte for lithium batteries. *Electrochim. Acta* **72**, 114-119 (2012).

5. Wu, H. B., Wei, S., Zhang, L., Xu, R., Hng, H. H., Lou, X. W. Embedding sulfur in MOF-derived microporous carbon polyhedrons for lithium-sulfur batteries. *Chemistry* **19**, 10804-10808 (2013).

6. Zhang, W., Qiao, D., Pan, J., Cao, Y., Yang, H., Ai, X. A Li^+-conductive microporous carbon-sulfur composite for Li-S batteries. *Electrochim. Acta* **87**, 497-502 (2013).

7. Lin, F., Wang, J., Jia, H., Monroe, C. W., Yang, J., NuLi, Y. Nonflammable electrolyte for rechargeable lithium battery with sulfur based composite cathode materials. *J. Power Sources* **223**, 18-22 (2013).

8. Li, Z. *et al.* Insight into the electrode mechanism in lithium-sulfur batteries with ordered microporous carbon confined sulfur as the cathode. *Adv. Energy Mater.* **4**, 1301473 (2014).

9. Zheng, S., Han, P., Han, Z., Zhang, H., Tang, Z., Yang, J. High performance C/S composite cathodes with conventional carbonate-based electrolytes in Li-S battery. *Sci. Rep.* **4**, 4842 (2014).

10. Zheng, S. *et al.* Copper-stabilized sulfur-microporous carbon cathodes for Li-S batteries. *Adv. Funct. Mater.* **24**, 4156-4163 (2014).

11. Li, G. *et al.* Sulfur/microporous carbon composites for Li-S battery. *Ionics* **21**, 2161-2170 (2015).

12. Luo, C., Zhu, Y., Wen, Y., Wang, J., Wang, C. Carbonized polyacrylonitrile-stabilized sesxcathodes for long cycle life and high power density lithium ion batteries. *Adv. Funct. Mater.* **24**, 4082-4089 (2014).

13. Wei, S., Ma, L., Hendrickson, K. E., Tu, Z., Archer, L. A. Metal-sulfur battery cathodes based on pan-sulfur composites. *J. Am. Chem. Soc.* **137**, 12143-12152 (2015).

14. Li, X. *et al.* Amorphous S-rich $S_{1-x}Se_x/C$ ($x \leq 0.1$) composites promise better lithium–sulfur batteries in a carbonate-based electrolyte. *Energy Environ. Sci.* **8**, 3181-3186 (2015).

15. Xu, Y. *et al.* Confined sulfur in microporous carbon renders superior cycling stability in Li/S batteries. *Adv. Funct. Mater.* **25**, 4312-4320 (2015).

16. Du, W. C., Zhang, J., Yin, Y. X., Guo, Y. G., Wan, L.J. Sulfur confined in sub-nanometer-sized 2 D graphene interlayers and its electrochemical behavior in lithium-sulfur batteries. *Chem. Asian J.* **11**, 2690-2694 (2016).

17. Fu, C., Wong, B. M., Bozhilov, K. N., Guo, J. Solid state lithiation-delithiation of sulphur in sub-nano confinement: a new concept for designing lithium–sulphur batteries. *Chem. Sci.* **7**, 1224-1232 (2016).

18. Wu, B., Chen, F., Mu, D., Liao, W., Wu, F. Cycleability of sulfurized polyacrylonitrile cathode in carbonate electrolyte containing lithium metasilicate. *J. Power Sources* **278**, 27-31 (2015).

19. Xu, Z. *et al.* Enhanced performance of a lithium-sulfur battery using a carbonate-based electrolyte. *Angew. Chem.* **55**, 10372-10375 (2016).

20. Niu, S. *et al.* Sulfur confined in nitrogen-doped microporous carbon used in a carbonate-based electrolyte for long-life, safe lithium-sulfur batteries. *Carbon* **109**, 1-6 (2016).

21. Hu, L. *et al.* Optimization of microporous carbon structures for lithium-sulfur battery applications in carbonate-based electrolyte. *Small* **13**, 1603533 (2017).

22. Hu, L., Lu, Y., Zhang, T., Huang, T., Zhu, Y., Qian, Y. Ultramicroporous carbon through an activation-free approach for Li-S and Na-S batteries in carbonate-based electrolyte. *ACS Appl. Mater. Interfaces* **9**, 13813-13818 (2017).

23. Zhu, Q., Zhao, Q., An, Y., Anasori, B., Wang, H., Xu, B. Ultra-microporous carbons encapsulate small sulfur molecules for high performance lithium-sulfur battery. *Nano Energy* **33**, 402-409 (2017).

24. Frey, M. *et al.* Easily accessible, textile fiber-based sulfurized poly(acrylonitrile) as Li/S Cathode material: correlating electrochemical performance with morphology and structure. *ACS Energy Lett.* **2**, 595-604 (2017).

25. Li, X. *et al.* Safe and durable high-temperature lithium-sulfur batteries via molecular layer deposited coating. *Nano Lett.* **16**, 3545-3549 (2016).

26. Gordin, M. L. *et al.* Bis(2,2,2-trifluoroethyl) ether as an electrolyte co-solvent for mitigating self-discharge in lithium-sulfur batteries. *ACS Appl. Mater. Interfaces* **6**, 8006-8010 (2014).

27. Busche, M. R., Adelhelm, P., Sommer, H., Schneider, H., Leitner, K., Janek, J. Systematical electrochemical study on the parasitic shuttle-effect in lithium-sulfur-cells at different temperatures and different rates. *J. Power Sources* **259**, 289-299 (2014).

28. Kim, H., Lee, J. T., Yushin, G. High temperature stabilization of lithium–sulfur cells with carbon nanotube current collector. *J. Power Sources* **226**, 256-265 (2013).

29. Kulisch, J., Sommer, H., Brezesinski, T., Janek, J. Simple cathode design for Li-S batteries: cell performance and mechanistic insights by in operando X-ray diffraction. *Phys. Chem. Chem. Phys.* **16**, 18765-18771 (2014).

30. Rosenman, A., Markevich, E., Salitra, G., Talyosef, Y., Chesneau, F., Aurbach, D. Facile synthesis and very stable cycling of polyvinylidene dichloride derived carbon: sulfur composite cathode. *J. Electrochem. Soc.* **163**, A1829-A1835 (2016).

31. Huang, J.-Q.*, et al.* Entrapment of sulfur in hierarchical porous graphene for lithium–sulfur batteries with high rate performance from -40 to 60°C. *Nano Energy* **2**, 314-321 (2013).

32. Ryu, H.-S.*, et al.* Discharge behavior of lithium/sulfur cell with TEGDME based electrolyte at low temperature. *J. Power Sources* **163**, 201-206 (2006).
